# Sound is essential for observers to accurately assess unsuccessful conversations

**DOI:** 10.1038/s41598-026-59805-z

**Published:** 2026-07-04

**Authors:** Raluca Nicoras, Rosa-Linde Fischer, Lauren V. Hadley, Graham Naylor, Karolina Smeds

**Affiliations:** 1 Hearing Sciences - Scottish Section, School of Medicine, University of Nottingham, New Lister Building Level 3, Glasgow Royal Infirmary, 10-16 Alexandra Parade, Glasgow, G31 2ER UK; 2 ORCA Labs, WS Audiology, Erlangen, Germany; 3 National Institute for Health and Care Research, Nottingham Biomedical Research Centre, Nottingham, UK; 4 ORCA Labs, WS Audiology, Lynge, Denmark

**Keywords:** Conversation success, Auditory-visual cues, Group conversations, Third-party observers, Neuroscience, Psychology, Psychology

## Abstract

Perceiving success in group conversations is central to social life, yet little is known about the extent to which the conversation success perceived by conversational partners aligns with that perceived by third-party observers. This study explores how third-party observers perceive conversation success across three sensory conditions (auditory-visual (AV), visual-only (V), and auditory-only (A)) to identify cues of which sensory domain signal perception of success. In two online experiments, older adults (57.1 years (SD = 6.1), *N* = 160 per experiment) watched and/or heard audio of 2-minute excerpts of previously recorded four-person conversations. Just as the original interlocutors had done themselves, observers provided continuous judgements of conversation success using a slider and an overall success rating after the conversation. Observers judged conversations in AV and V conditions (Experiment 1) and AV and A conditions (Experiment 2). Across both experiments, observers’ overall ratings generally aligned with interlocutors’ overall success ratings, showing that success can be assessed without active participation. However, for less successful conversations, observers overestimated success when deprived of auditory information (V condition) but not when deprived of visual information (A condition). These findings suggest that auditory cues provide the most critical information for assessing conversation failure, while visual cues alone may mislead observers.

## Introduction

Conversations vary in how successful they feel to those involved, and being able to judge their success is important for understanding social interaction. In everyday life, people monitor other people’s conversations to infer how interlocutors are feeling and how well an interaction is progressing. This can occur across a range of contexts, from leading group discussions at work to family chats around the dinner table. These judgments can guide interaction behaviour, including when to intervene to support someone who may be struggling.

Perceived success has been found to depend on whether ideas are exchanged smoothly, whether participants feel included, and whether communication difficulties are addressed^1^. Visual cues such as gestures and facial expressions, and auditory cues such as prosody, semantic content, and turn-taking, may contribute to perceptions of conversational success.

Observers are often able to judge social interactions from limited information^2^, yet the accuracy of these judgments depends on which cues are available. Evidence for this comes from studies examining third-party observer judgments in situations where auditory information is unavailable and judgments must be based on visual cues alone, or visual information is unavailable and judgments rely only on auditory cues. Evidence from visual-only studies suggests that observers can make surprisingly accurate social judgments even in the absence of auditory information. When the auditory signal is removed, laboratory studies show that observers can distinguish threat from teasing in interactions solely from body movements^3^. Similarly, silent videos, even as brief as ten seconds, allow observers to rate the likability of interlocutors, their judgements being shaped by nonverbal synchrony^4^.

In other domains, such as music, listeners could differentiate the size of sung melodic intervals based only on singers’ facial expressions and head movements in the absence of an auditory signal, suggesting that visual cues can convey information about pitch relationships that can be interpreted by viewers^5^. Moreover, it seems that the visual impact can be so strong that it can override the auditory information. For example, it has been shown that listeners can guess music competition winners based on performers’ visual behaviour (e.g., movements) rather than on how they sounded^6,7^. In music performance, emotions are a central aspect of the listener experience, and visual cues have been shown to contribute substantially to this process. For example, when comparing emotions elicited in different modalities (live auditory-visual music performance, recorded auditory-visual, recorded auditory-only, and recorded visual-only), one study found that some emotional responses were more strongly associated with visual information than with auditory information alone^8^. This finding suggests that, at least for some emotions, the visual input may be a more potent conveyor of the performance’s emotional impact than the auditory input on its own. Although these findings arise in a domain often assumed to be primarily auditory, they highlight the extent to which visual information can shape observers’ perception.

However, in conversations, auditory information conveys semantic content, prosody, rhythm, and turn-taking patterns that show how meaning and coordination unfold^9–11^. For example, Wynn et al. (2022) found that when two people’s speaking rates were synchronised, third-party listeners judged their conversations as higher in quality than when the interlocutors’ speaking rates were not synchronised. Auditory information could therefore provide rich cues to conversational coordination and success, yet some aspects of listening difficulty can remain inaudible to third-party observers. Conversational effort, for instance, may be heard when communication breaks down and interlocutors ask for clarification or repetition^13^. However, Winn & Teece (2025) showed that third-party listeners could not accurately distinguish between speech produced under high versus low communication-effort conditions when judging voice recordings, indicating that communication effort is not always audible to others.

Previous work has shown that conversation success is multifaceted, reflecting elements that may be conveyed through both visual and auditory channels, including ease of listening, engagement, conversational flow, and positive emotions^1^. However, it remains unclear whether observers’ ratings of conversation success align with those of the original interlocutors nor whether their ratings are influenced by sensory access. Visual-only (V) access preserves smiles, nods, gaze, etc., but removes prosody and lexical content, which might lead to misinterpretation. Auditory-only (A) access preserves content and vocal timing but removes body language that can signal inclusion or (dis)engagement. Although behaviour can be categorised by sensory modality, many functionally related cues operate across modalities, meaning that removing one modality can obscure how these cues jointly convey social meaning^15^. Therefore, an auditory-visual (AV) condition provides a baseline in which these cues remain integrated, and comparing AV against V and A allows us to isolate what each stream uniquely contributes to success judgments. This simulates a limitation that also constrains artificial systems, such as hearing devices and voice-based agents, which typically capture audio but not video. While such systems do not assess conversational success explicitly, their ability to adapt, support, or respond appropriately may depend on cues that are distributed across modalities. Establishing how judgments may degrade without vision or audio, could help in the setting of design targets for assistive devices. These findings could also have implications for other domains in which communicative effectiveness depends on auditory-visual integration, including broadcast media (e.g., television versus radio) for political debates^16,17^ and consumer research^18^.

Most research on conversation success has examined the perspective of active interlocutors^19,20^. Less is known about whether third-party observers, individuals not taking part in the interaction, can accurately judge whether a conversation is going well. Previous studies have shown that third-party observers can assess communication behaviour from audio-visual recordings^21,22^. However, to our knowledge, the accuracy of observer judgments has not been tested against interlocutors’ self-assessments of conversation success. Hence, we aim to address two questions in this paper. First, how well do observers’ ratings match the judgments of the original interlocutors? Second, how much do different sensory cues influence observers’ judgments? In natural conversation, both auditory and visual cues are available, but each may contribute differently to observers’ ratings of conversation success.

Many of the previous studies have focused on observers judging dyadic interactions^3,4,14,23^. However, group conversations account for a substantial proportion of everyday social interactions^24,25^, occurring frequently in settings such as family gatherings, workplaces, and social groups. Compared with dyadic exchanges, group conversations place greater demands on participants’ attentional resources, as interlocutors must monitor multiple speakers, manage turn-taking, and track parallel conversational cues. These characteristics make group conversations particularly relevant for studying how third-party observers judge conversation success, as recognising when participants may be struggling or require support can be inherently more complex in multi-party interactions.

To address these gaps, we conducted two online experiments with older adult observers who rated short excerpts of four-way group conversations (also involving older adults). In Experiment 1, observers judged conversations presented in AV and V conditions. In Experiment 2, observers judged the same conversations in AV and A conditions. The excerpts were selected so that half came from conversations the original interlocutors rated as ‘successful’ and half from those they rated as ‘unsuccessful’. In both experiments, observers used a slider to continuously rate the success of the ongoing conversation. They then gave an overall rating of the conversation success at the end of each video followed by an open-ended explanation of their judgement.

We hypothesised that observers would reproduce the success ratings of the original interlocutors in the AV conditions, demonstrating that conversation success can be accurately assessed from third party observers’ perspective (H1). We also expected success rating accuracy to decrease in both single-sensory conditions (H2). However, given the evidence showing that visual cues can strongly shape observers’ judgements^3–8^, while auditory-only information may fail to convey relevant behaviours such as communication effort^14^, we expected that removing visual information (A condition) would impair success rating accuracy more than removing auditory information (V condition) (H3). In the results section we report observers’ ratings of conversation success from both experiments. We also include an exploratory word-frequency analysis of the open-ended responses to shed light on the cues observers considered when evaluating conversation success.

## Results

### Overall ratings of conversation success – observers vs. interlocutors

**Fig. 1:**
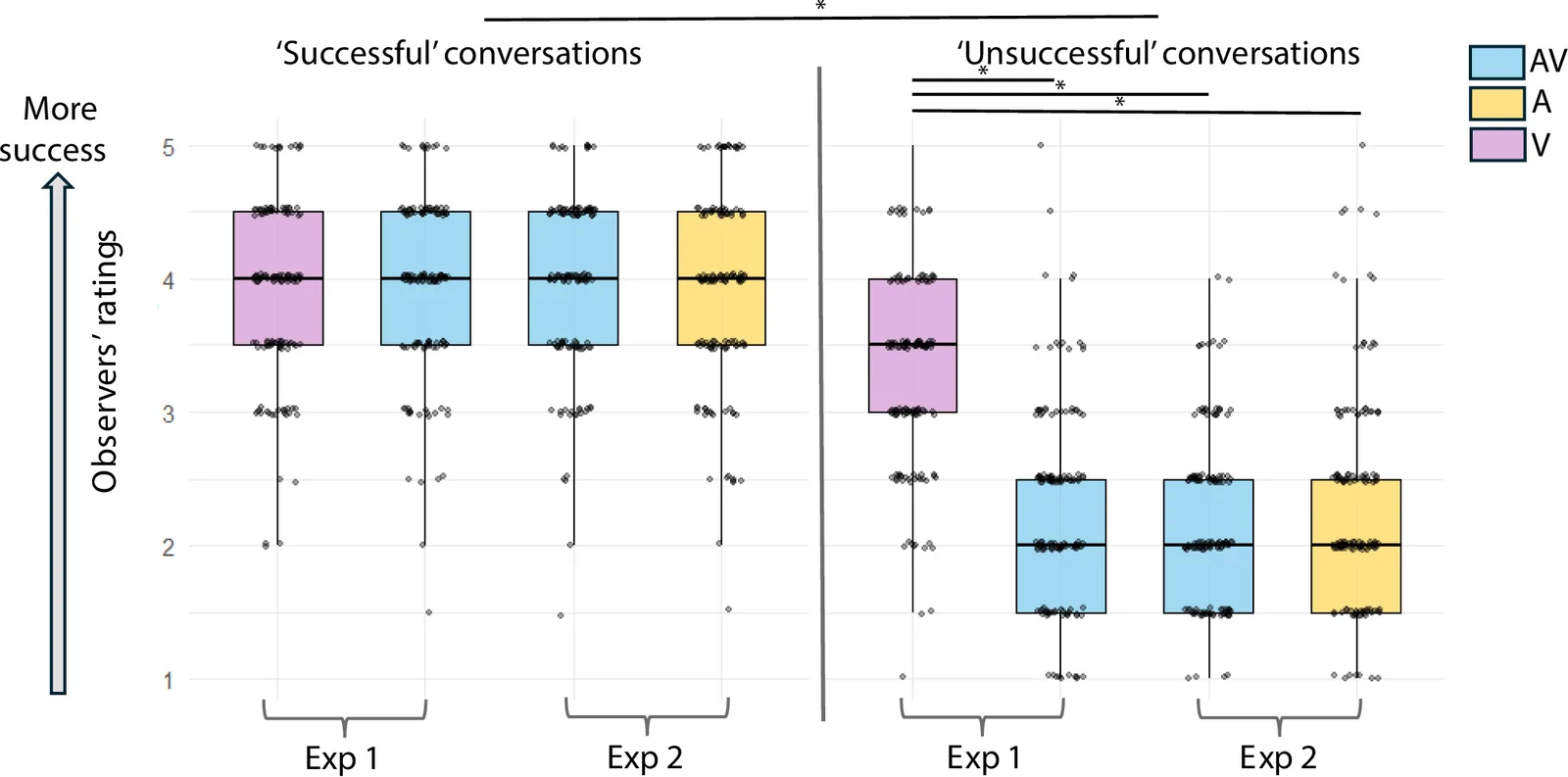
Overall ratings of conversation success across sensory conditions. Boxplots show observers’ post-observation ratings of conversation success (1–5 scale) for ‘successful’ (left) and ‘unsuccessful’ (right) conversations (as judged by the original interlocutors) in each sensory condition: auditory–visual (AV), auditory-only (A), and visual-only (V), grouped by experiment (Exp 1 and Exp 2). Boxes represent the interquartile range (IQR), with the horizontal line in the boxes showing the median. Whiskers extend to 1.5 × IQR. Individual points represent individual observers’ ratings for each condition, averaged across the two videos they watched. Asterisks indicate statistically significant differences between conditions.

To assess whether observers reproduced interlocutors’ ratings of ‘successful’ and ‘unsuccessful’ interactions, Wilcoxon signed-rank tests (for within-experiment comparisons) and Mann–Whitney U tests (for between-experiment comparisons) compared ratings across conditions. These analyses were conducted at the condition level: because each observer experienced two recordings corresponding to the same condition (modality x ‘un/successful’), ratings were averaged across these recordings to obtain a single score per observer and condition for the statistical analyses. Observers rated ‘successful’ conversations significantly higher than ‘unsuccessful’ ones in all conditions (auditory-visual Experiment 1: Z = 10.903, *p* < .001; Experiment 2: Z = 10.865, *p* < .001; auditory-only Z = 10.854, *p* < .001; and visual-only Z = 8.501, *p* < .001), confirming our first hypothesis (H1). The results showed that third-party observers’ ratings followed the original interlocutors’ ratings (Fig. 1).

For ‘successful’ conversations, ratings did not differ significantly across sensory conditions, indicating that observers judged ‘successful’ interactions similarly regardless of whether auditory and/or visual information was available. In contrast, for ‘unsuccessful’ conversations, ratings in the visual-only condition were significantly higher than in all other sensory conditions (compared to the auditory–visual condition of Experiment 1 (Z = 9.206, *p* < .001); compared to the auditory–visual condition of Experiment 2 (Z = 11.350, *p* < .001); and compared to the auditory-only condition (Z = 10.509, *p* < .001)), and the median rating was in the successful region (above 3). While this pattern partially supports our hypothesis that rating accuracy would decrease when only a single sensory modality is available (H2), it contradicts our expectation that accuracy would be lower in the auditory-only condition than in the visual-only condition (H3).

### Continuous ratings of conversation success – comparing modalities

**Fig. 2:**
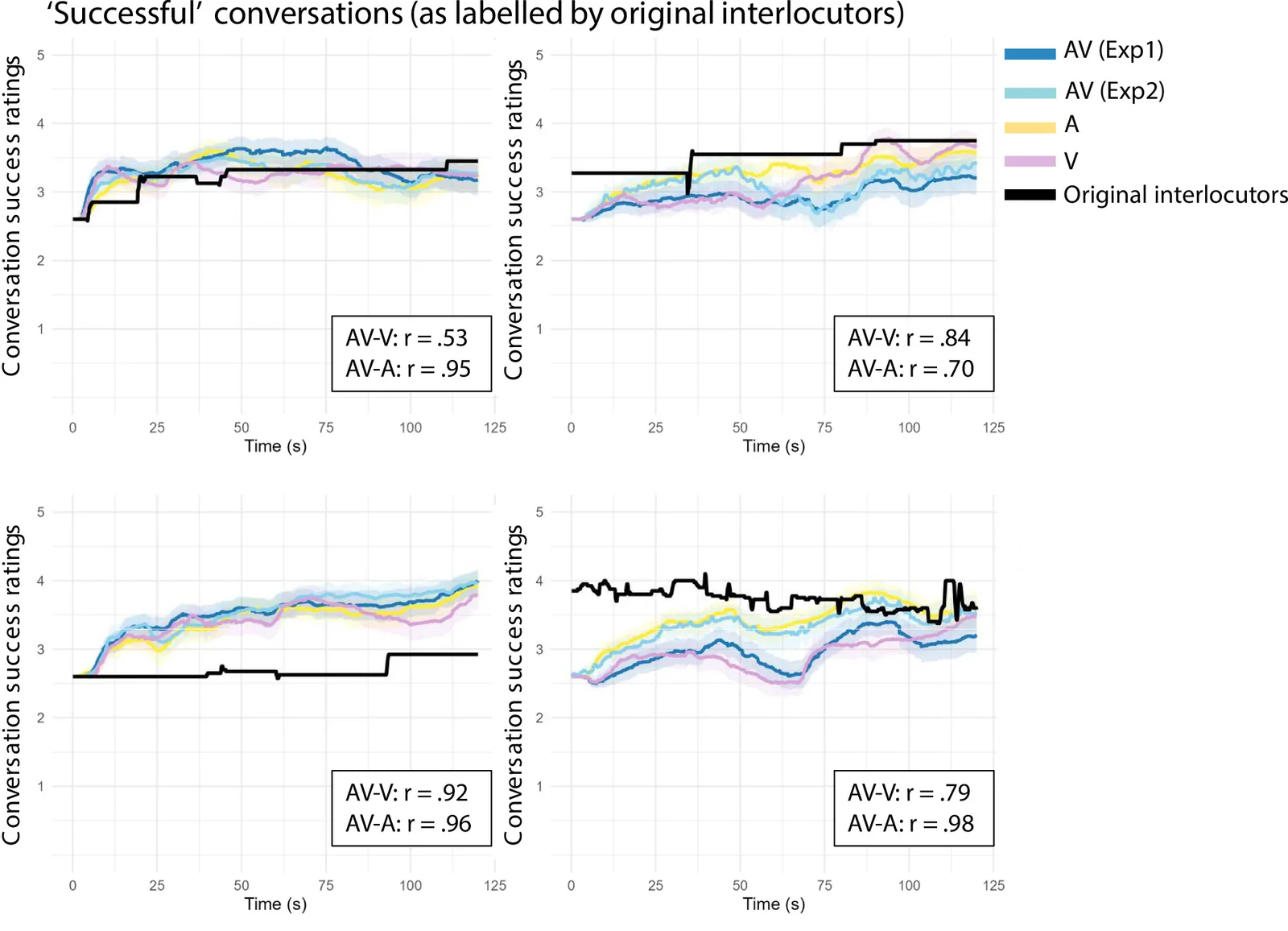
Slider ratings of conversation success over time for four individual conversations labelled as successful by the original interlocutors (1–5 scale on y-axis; seconds on x-axis). Coloured lines show condition means across observers; shaded bands show 95% CIs. Black lines show mean across original interlocutors (*n* = 4/conversation). Annotated values are Spearman correlations (r) between the two time-series coming from the same experiment (V with AV Exp1 and A with AV Exp2) per video. All correlations are statistically significant (*p* < .05).

**Fig. 3:**
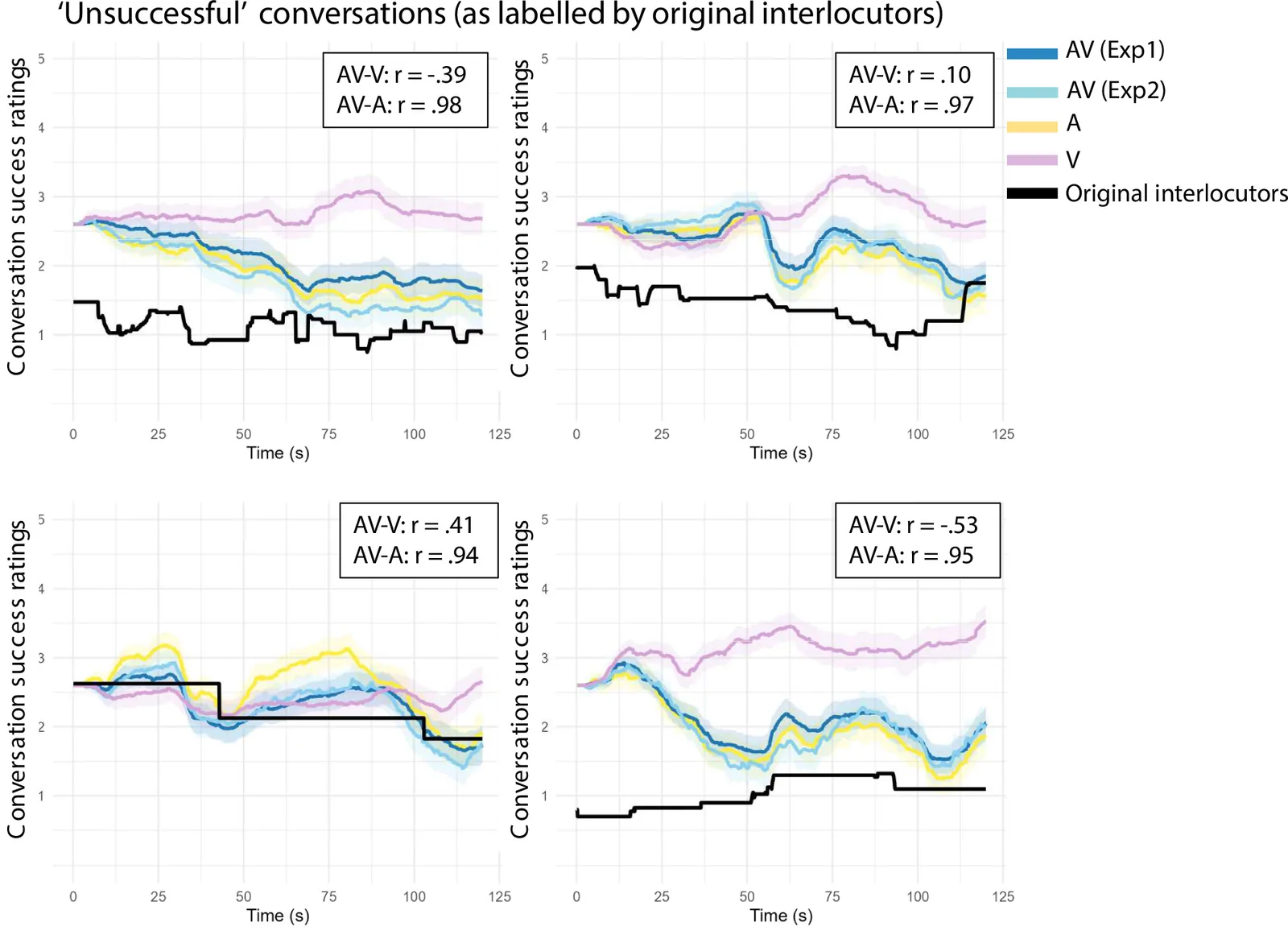
Slider ratings of conversation success over time for four individual conversations labelled as unsuccessful by the original interlocutors (1–5 scale on y-axis; seconds on x-axis). Lines show condition means across observers; shaded bands show 95% CIs. Black lines show mean across original interlocutors (*n* = 4/conversation). Annotated values are Spearman correlations (r) between the two time-series coming from the same experiment (V with AV Exp1 and A with AV Exp2) per video. All correlations are statistically significant (*p* < .05).

Correlations between time-series of slider ratings across sensory conditions (AV vs. V in Experiment 1; AV vs. A in Experiment 2) show that the effects of modality on observers’ moment-by-moment judgments followed the same pattern as their overall ratings reported above. These analyses were conducted at the level of individual conversations: each panel in Figs. 2 and 3 represents a different conversation, with the same conversation presented to different observer groups in AV, V, or A conditions.

For ‘successful’ conversations, correlations were high across AV-V and AV-A modality pairs (all *r* > .53, *p* < .05), indicating that observers tracked conversation success similarly regardless of whether only auditory or visual information was available (Fig. 2). For ‘unsuccessful’ conversations, correlations remained high between AV and A conditions but were weak or negative between AV and V conditions, revealing a larger divergence in slider patterns (Fig. 3).

### Exploratory word-frequency analysis

Across all conditions, the most frequent umbrella terms provided by observers in the free-text responses were “talking/speaking/saying” (*n* = 783), “hearing” (*n* = 681), “laughing” (*n* = 425), and “engaging” (*n* = 415) (Table 1). These were consistent in AV and A conditions, but not in V conditions. In V conditions, frequent terms included “smiling” (*n* = 189), “talking/speaking/saying” (*n* = 126), “engaging” (*n* = 99), “nodding” (*n* = 93), and “dominating” (*n* = 40). The words used by observers can appear in negative form (“talking” → “not talking”) or in phrases with negative connotation (e.g., “talking over each other”). Manual verification showed that, for example, for the term “talk” and its variants, most occurrences in ‘unsuccessful’ conversations referred to non-affirmative or disrupted forms of talk (e.g., absence of talk, overlapping talk). Full details are reported in Appendix A.

**Table 1:**
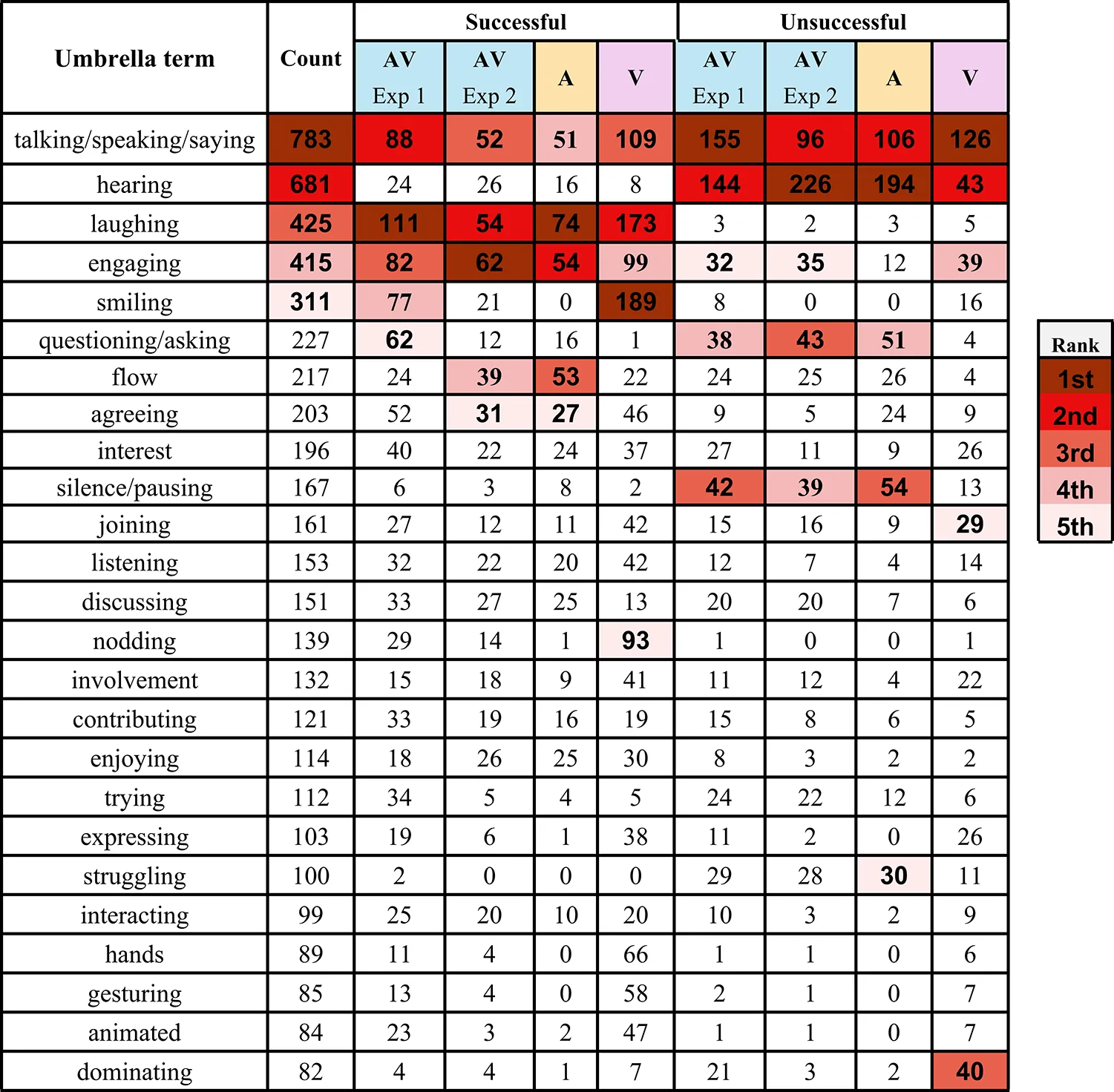
Frequency of descriptive terms used by observers across sensory conditions. Box shading shows the top five ranks per condition column (dark red = 1st, to light red = 5th ). The ‘successful’ and ‘unsuccessful’ label reflects interlocutors’ ratings.

## Discussion

The present study examined how access to auditory and visual information shapes third-party judgments of conversation success. Across two experiments, observers accurately assessed interactions that were previously judged as ‘successful’ and ‘unsuccessful’ by interlocutors, demonstrating that conversational success can be inferred without active participation. Nevertheless, the accuracy of these judgments depended on sensory access. In auditory-visual and audio-only conditions, the observers’ ratings aligned with those of the interlocutors. However, when auditory information was removed, observers rated ‘unsuccessful’ conversations as more successful than the original interlocutors did, and their continuous ratings while watching the conversation diverged from those made when sound was available. This indicates that auditory information provides useful clues for judging when a conversation is unsuccessful.

Prior research highlights the significance of visual cues in communication^26^ and individuals’ ability to derive meaning from visual signals alone^27,28^. For example, studies have shown that people can discern intentions such as rejection or acceptance from nonverbal behaviour^27^ and can recognise emotions from muted videos or static images^29^. Moreover, observers’ interpretations of behaviour can be shaped by the particular visual cues present. For example, it has been shown that when addressees display signals such as smiles, brow raises, or dimples during response planning, observers expect affirmative responses, whereas gaze aversion, brow frowns, nose wrinkles, or squints lead observers to anticipate negative responses^28^. While observers can use facial and bodily movements to anticipate or infer the course of an interaction^30^, these cues alone may not reliably convey whether an exchange is going well. The current study confirms this limitation: in the absence of auditory information, observers relied on visible cues such as smiling, which may signal positive affect but not necessarily conversation success.

Contrary to our expectations, auditory information appears to provide more reliable cues for judging whether conversations are going well or poorly. Acoustic features such as pitch, rhythm, and timing can reveal coordination and alignment between speakers, which have been linked to judgments of conversational quality^12^. Auditory information also carries the verbal content of speech, offering contextual information that can alter how behaviours are interpreted. Listeners integrate what is said with how it is said when forming judgments about an interaction^31,32^. Hearing the verbal content can clarify whether a behaviour signals agreement, disagreement, or humour. For instance, an interlocutor’s head shake and grimace might appear to convey disgust or rejection and lead observers to judge the exchange as unsuccessful. When accompanied by the audio, observers could hear the interlocutor say “Eww, I don’t like cheese!”. This shows how the same behaviour can be reinterpreted as humorous, illustrating how access to verbal content changes observers’ understanding of interactional meaning.

The exploratory word-frequency analysis confirmed the limitations of the visual-only condition. In ‘unsuccessful’ conversations, observers did not recognise when participants were asking questions such as requests for repetition, which can signal listening difficulty and indicate a decline in conversation success. Although observers frequently referred to talking (*n* = 126), without access to the speech content they could not identify that many of these utterances were attempts to clarify misunderstanding (they mentioned “flow” and “questioning” only 4 times each). Instead, they appeared to interpret visible speech movements as signs of “involvement” (*n* = 22, compared with fewer mentions when other modalities were available for the ‘unsuccessful’ conditions). Words reflecting communicative difficulty, such as “struggling” or “trying”, were also mentioned less often than for other conditions, suggesting that signs of effort were not easily perceived without sound. However, one aspect was noted more often in the visual-only condition of ‘unsuccessful’ conversations than in the others: dominance. Observers frequently commented that the conversations were dominated by one or two people (*n* = 40), indicating that visual information can still reveal some aspects of conversational imbalance even when auditory cues are unavailable.

### Strengths and limitations

An important strength of this study is the direct link between observers’ and interlocutors’ ratings of conversation success, offering a valuable reference for assessing the accuracy of observers’ judgments. In addition, observers provided ratings both during and after watching the conversations, allowing visualisation of time points in the videos where perceptions of success diverged between conditions. These findings also open avenues for future work, including segment-by-segment analyses of how judgments evolve over time and investigations of the behavioural sequences that drive these changes.

One limitation is that observers viewed only two-minute excerpts from six-minute conversations. It is therefore possible that the original interlocutors’ ratings of success were influenced by events occurring outside the selected excerpts. However, previous work using the same dataset^19^ showed that interlocutors’ continuous ratings of conversation success were highly correlated with their overall post-conversation ratings, which were used to define the successful and unsuccessful labels of segments used as stimuli in this study. Nevertheless, the present findings indicate that even brief excerpts of conversation can contain meaningful cues about conversational success, supporting previous research showing that social judgments can be formed from limited samples of behaviour^2^.

One potential confounding factor is the way observers played back the recordings. Although observers were instructed to use a computer and headphones, we could not verify the exact screen size, audio quality, or listening volume used. Variability in these factors may have influenced visibility, intelligibility and, consequently, observers’ ratings of conversation success.

The auditory-only condition in this study contained both lexical and non-lexical information. As these sources of information were available together, the present design does not allow conclusions about whether observers’ judgment accuracy was driven primarily by lexical content, prosodic cues, or their combination. Future work could separate these components, for example by isolating paralinguistic cues, to examine the relative contribution of each auditory information stream to judgments of conversation success.

The present findings may have implications for the design of technologies intended to support communication. In particular, the results suggest that auditory information plays a critical role in judgments of conversational success and should therefore be prioritised in systems designed to facilitate conversational interactions. However, these implications remain speculative, as the present study examined subjective human judgments rather than the performance of assistive technologies. Further research is needed to identify objective behavioural indicators of conversational success and to determine how such information could be detected and used by assistive systems.

## Conclusion

This study showed that conversation success can be judged accurately by third-party observers in the auditory-visual condition. However, the accuracy of these judgments depends on sensory access. When only auditory cues were available, observers’ ratings matched closely those of the interlocutors who took part in the conversations. In contrast, when only visual cues were available, observers overestimated success in ‘unsuccessful’ conversations. This suggests that auditory cues suffice, but visual cues do not, for detecting communication challenges. Analyses of word frequencies further confirmed that observers in the visual-only condition were not able to identify cues such as questioning to convey difficulty or silence as a signal of conversation breakdown. Without access to the auditory context, they often interpreted talking as a sign of engagement, even though it could equally reflect communication challenges such as requests for repetition. Nevertheless, visual cues offered some insight into the structure of the interaction, including patterns of participation and conversational dominance. These findings provide a foundation for understanding how people interpret social interactions when sensory information is limited.

## Materials and methods

### Participants

**Fig. 4:**
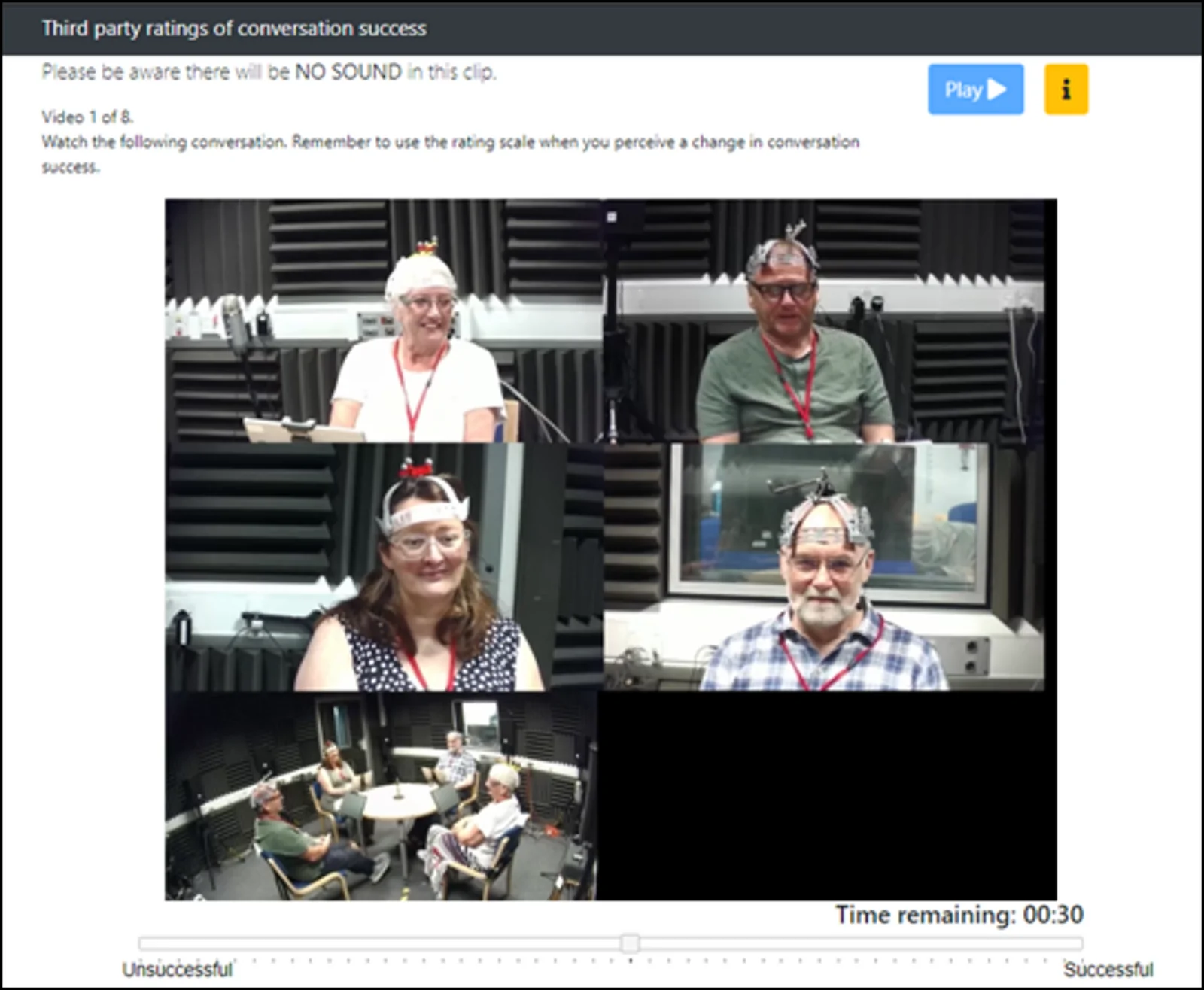
Screenshot of NOTE survey showing the observers’ view. The slider which observers used to rate conversations can be seen below the video.

A total of 320 observers were recruited through the Prolific platform (www.prolific.com). Participants were required to be aged between 50 and 75 years, fluent English speakers residing in the United Kingdom, self-report normal hearing, and be able to use a computer and headphones. The age criterion was selected to broadly match the age range of the interlocutors featured in the stimuli. The remaining criteria were selected to minimise potential biases related to language proficiency, hearing difficulties, or technical challenges. They were divided into two independent samples of 160 observers per experiment: Experiment 1 (auditory–visual vs. visual-only conditions) and Experiment 2 (auditory–visual vs. auditory-only conditions). The mean age across experiments was 57.1 years (SD = 6.1), with a balanced gender distribution (162 females, 158 males). Observers were compensated at a rate of £10 per hour and completed the task in 36 min on average. All observers provided informed consent online. Ethical approval was obtained from the West of Scotland Research Ethics Committee (18/WS/0007) and NHS Greater Glasgow and Clyde R&D (GN18EN094), and all procedures performed are in accordance with the Declaration of Helsinki (World Medical Association, 2013). The informed consent for the release of identifying images for commercial uses, such as an online open-access publication, was obtained from all interlocutors depicted in Fig. 4.

### Procedures

Observers completed the experiment online using the Nottingham Online Testing and Experimental platform (NOTE). After providing consent, each observer viewed eight two-minute video excerpts of four-person group conversations that had been recorded in a previous study^33^. The original interlocutors were older adults (M = 62.1 years, SD = 6.7). Each video was selected from a six-minute original interaction involving people with normal and impaired hearing. Conversations were classified as successful or unsuccessful based on the mean post-conversation success ratings provided by the original interlocutors. Four conversations with a mean rating above 3 were labelled ‘successful’, and four with a mean rating below 3 were labelled ‘unsuccessful’. Segments were chosen to meet the following criteria: (a) no explicit verbal or gestural disclosure of hearing impairment; and (b) the excerpt began at a complete utterance rather than mid-speech. Acoustic characteristics were not used as selection criteria. Although original interlocutors with hearing loss were wearing hearing aids in two of the eight selected videos, observers were not provided with any information regarding the hearing status or hearing aid use of the interlocutors to avoid bias and the hearing aids were not visible in the selected excerpts. During the original conversations, each interlocutor had a tablet positioned in front of them on the table, which was used to continuously rate conversation success. These tablets are visible in the videos shown to observers; however, the screens were blurred (Fig. 4). Although occasional hand movements may have been visible, observers were unable to determine rating values, rating direction, or that the screens displayed conversation-success ratings, and they were not informed of the tablets’ purpose.

Each observer saw one version of each of the videos, with sensory condition varying by experiment. In Experiment 1, half of the videos were presented in the auditory-visual (AV) condition and half in the visual-only (V) condition. In Experiment 2, half were auditory–visual (AV) and half auditory-only (A). Video order and condition assignment were counterbalanced across observers.

### Stimuli description

The stimuli consisted of eight video recordings of four-person group conversations captured in a laboratory environment. Each stimulus displayed five synchronised video streams: four portrait views showing the individual interlocutors and one wide-angle view showing the group interaction (Fig. 4). This arrangement allowed observers to view participants’ faces, head movements, and upper body behaviours (e.g., hand gestures). Audio was recorded using four microphones, one per interlocutor. The four audio channels were subsequently combined into a single audio track and synchronised with the video recording. In the auditory-only condition, observers listened to the audio recording without access to the video, whereas in the visual-only condition, observers viewed the video recording without audio. All videos were recorded using the same camera configuration, room setup, seating arrangement, and display format, thereby minimising potential confounding factors unrelated to conversational success.

### Task

During each video, observers continuously rated perceived conversation success using a slider ranging from “unsuccessful” (0) to “successful” (5), however the scale points were hidden on the slider (Fig. 4). Immediately after each video, they provided an overall rating on the same five-point Likert scale (1 = unsuccessful, 5 = successful) the original interlocutors had used and responded to two open-ended questions: They then responded to two open-ended questions: “During the video, there may have been moments when you felt the conversation was progressing well. What specific features or actions led you to perceive these as successful instances in the conversation?” and “Conversely, there may have been moments when you felt the conversation was not going as well. What features or actions caused you to perceive these as less successful instances in the conversation?.

### Data analysis

#### Observers’ overall ratings of conversation success

The normality of the conversation success rating (Likert 1–5) distributions was examined using the Shapiro–Wilk test, which confirmed that the data were not normally distributed (*p* < .05). Together with the ordinal nature of the Likert scale, this justified the use of non-parametric tests. Although each observer rated eight videos (two per condition), ratings for the two videos within each condition were averaged to obtain one mean value per observer per condition. This resulted in a dataset of 160 observations per condition (Successful/Unsuccessful Auditory-visual, Successful/Unsuccessful Visual only in Experiment 1; Successful/Unsuccessful Auditory-visual, Successful/Unsuccessful Auditory only in Experiment 2).

Observers’ overall ratings of conversation success were then compared across sensory conditions (AV vs. V in Experiment 1 and AV vs. A in Experiment 2) and conversation types (‘successful’ vs. ‘unsuccessful’). For each experiment, Wilcoxon signed-rank tests were used to compare ratings within the same experiment (‘successful’ versus ‘unsuccessful’ conversations within conditions for each experiment). Mann–Whitney U tests were used to compare ratings of the same conversation type between experiments (e.g., Experiment 1 ‘unsuccessful’ V vs. Experiment 2 ‘unsuccessful’ A). To control for multiple comparisons, Bonferroni correction was applied to the resulting p-values.

#### Continuous ratings of conversation success

Slider data were sampled at 10 datapoints per second and averaged across observers for each video. To assess correspondence between real-time judgments across sensory conditions, Spearman correlations were computed between the mean time-series of AV and V conditions in Experiment 1, and between AV and A conditions in Experiment 2. Bonferroni correction was also applied to the correlation analyses to adjust for multiple comparisons. Correlation coefficients (r) are reported in the Results section (Figs. 2 and 3).

#### Word-frequency analysis

Open-ended responses were analysed using NVivo 15 to explore which behavioural descriptors observers used most frequently across conditions. Words sharing the same stem (e.g., smile, smiling, smiles) were grouped together, and frequency lists were generated from most to least frequent.

To focus the analysis on observable conversational behaviours, words that did not describe a behaviour were removed. For example, in the phrase “Participants seemed engaged”, only “engaged” was retained, whereas “participants” and “seemed” were excluded because they do not refer to specific conversational behaviours. Behavioural terms (e.g., smiling, talking) and descriptive words closely tied to conversational dynamics (e.g., engaging, flowing) were retained. Certain semantically related words were combined into umbrella categories, such as speaking/talking/saying and silence/pausing. For some stems, additional restrictions were applied to refine meaning: “look” was limited to “look at” to capture gaze-directed actions rather than descriptive expressions (e.g., “looking bored”); “listen” excluded “listener(s)”; and “point” excluded “point(s)”, with only “pointing at” retained as the noun form “point(s)” was commonly used in a temporal or abstract sense (e.g., “at this point in time”), rather than to describe a physical pointing action.

From the resulting list, the 25 most frequent behaviour and descriptor umbrella terms across conditions were selected. These umbrella terms were then compared across sensory conditions (auditory–visual, auditory-only, and visual-only) and conversation types (successful vs. unsuccessful) to identify condition-specific patterns in observers’ descriptions. For each sensory condition and conversation type, we ranked the top five terms (1 = most frequent); see Table 1 in the Results. We also manually checked each of these terms to determine whether it was used as an affirmation (e.g., “talking”) or as a negation or in a phrase with negative meaning (e.g., “not talking”, “talking over each other”). An expanded version of Table 1, reporting for each term the percentage of occurrences coded as negation/negative meaning, is provided in Appendix A.

## Figures and Tables

**Table 2:**
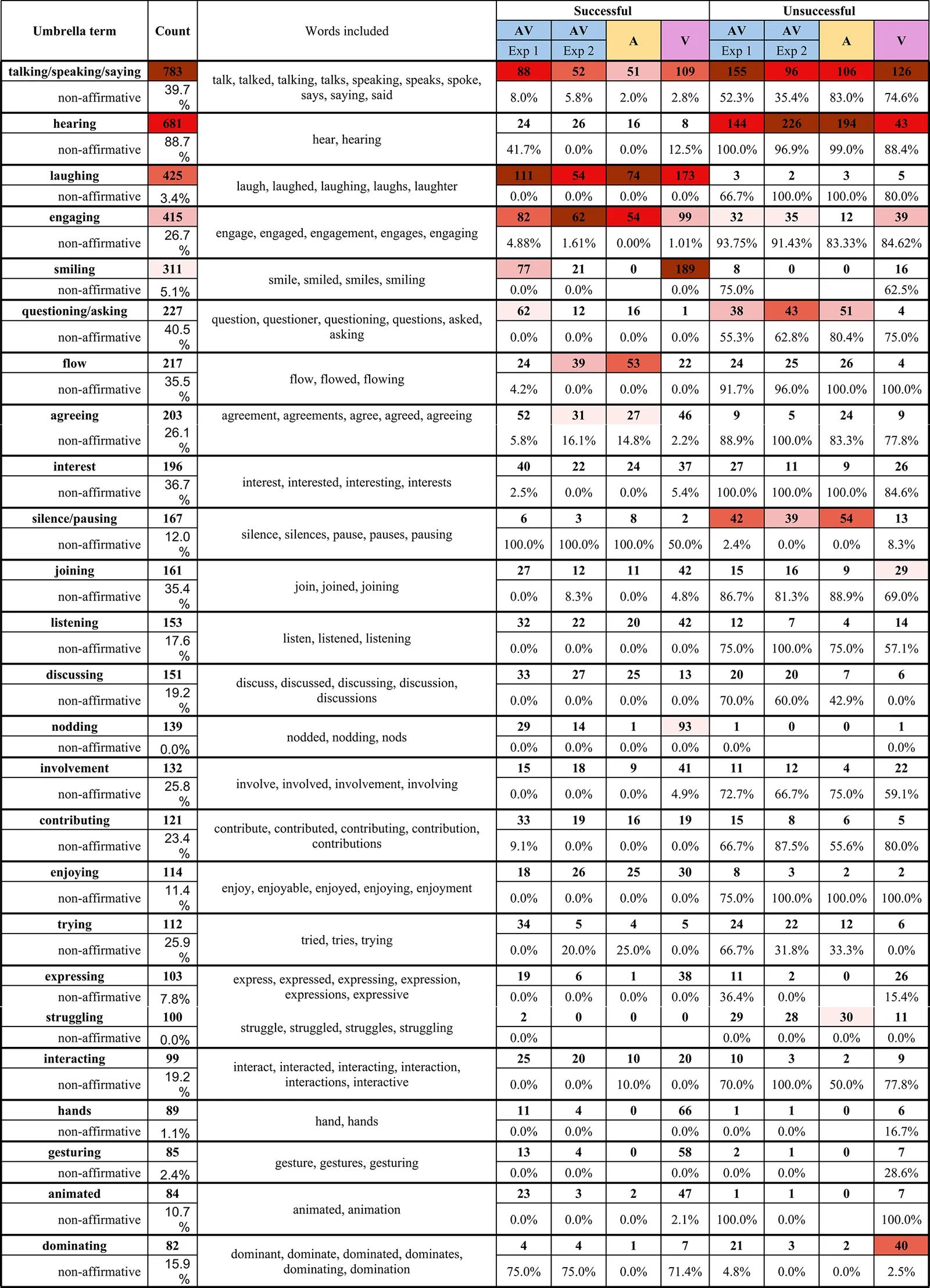
Full list of umbrella terms with included words, total counts, and counts by sensory condition (AV, A, V) and conversation type (successful, unsuccessful). The “non-affirmative” rows report the percentage of occurrences used as negations or with negative meaning (e.g., “not talking”, “talking over each other”). Box shading shows the top five ranks per condition (dark red = 1st, to light red = 5th ). * Terms whose affirmative use typically carries or can carry negative connotations depending on the context (e.g., “trying hard”).

## Data Availability

The datasets generated and analysed during the current study are available from the corresponding author on reasonable request.

## Appendix A

See Table 2 here.
